# Declarations of conflicts of interest from the editors of the *Journal of Global Health* – 2019

**DOI:** 10.7189/jogh.09.010102

**Published:** 2019-06

**Authors:** Ana Marušić, Igor Rudan, Harry Campbell

We continue the practice of transparency of potential competing interests for us as editors of the *Journal of Global Health* [1], following the International Committee of Medical Journal Editors (ICMJE) Recommendations for the Conduct, Reporting, Editing, and Publication of Scholarly work in Medical Journals [2]. As we expect from our authors and reviewers to declare their possible conflicts of interest in relation to the manuscript under consideration for publication in the journal, we also declare our commitments and relations that may influence the editorial review process. When one of the decision-making editors submits an article to the *Journal*, it is reviewed independently from the editor, who has no access to or influence on the review process and editorial decision-making. This is always indicated in the Competing Interests declaration at the end of the published article.

We declare below the following conflicts of interest for the current year.

## PROFESSOR ANA MARUŠIĆ, MD, MS, PHD

### Personal and organizational

Prof. Marušić is fully employed by the University of Split School of Medicine, where she holds a tenured position as Professor and Chair of the Department of Research in Biomedicine and Health. She receives funding support for research from the Croatian Science Foundation and the European Commission under the Horizon 2020 Programme. She serves as an external expert for the European Commission and Council of Europe. She is also a Visiting Professor at the NTU Lee Kong Chian School of Medicine, Singapore.

### Journal

Prof. Marušić holds a position as the Editor in Chief. She occasionally reimburses small expenses related to Journal’s work, including travel and consumables.

### Honorary positions

Honorary Professor, College of Medicine and Veterinary Medicine, University of Edinburgh, Edinburgh, Scotland, UK;
Visiting Professor, School of Medicine, University of Sao Paulo, Sao Paulo, Brazil:
Past President, European Association of Science Editors;
Steering Group, EQUATOR Network;
Co-Chair, Cochrane Scientific Committee;
Research Coordinator, Cochrane Croatia;
Advisory Board Member, the Balkan Medical Journal.

## PROFESSOR IGOR RUDAN, MD, DSC, PHD, MPH, FRSE

### Personal and organizational

Prof. Rudan is employed by the University of Edinburgh, where he holds a position as Professor and Co-Director of the Centre for Global Health Research. He is also co-Director of the WHO Collaborating Centre in Population Health Research and Training. He currently receives research funding support from the Gates Foundation.

### Journal

Prof. Rudan holds a position as the co-Editor in Chief. He occasionally reimburses small expenses related to Journal’s work, including travel and consumables.

### Paid positions

Prof. Rudan has numerous technical advisor appointments to WHO, UNICEF and the World Bank.

## PROFESSOR HARRY CAMPBELL, MD, FRCPE, FFPH, FRSE

### Personal and organizational

Prof. Campbell is employed by the University of Edinburgh, where he holds a position as Professor, Co-Director of the Centre for Global Health Research and acting Dean of Molecular, Genetic and Population Health Sciences in the College of Medicine and Veterinary Medicine. He is also co-Director of the WHO Collaborating Centre in Population Health Research and Training and co-Director of the NIHR Global Respiratory Health Unit. He currently receives research funding support from the European Commission (Innovative Medicines Initiative), WHO, UK NIHR, Cancer Research UK, UK MRC, Sanofi (for work on specific GI and respiratory infections) and the Gates Foundation.

### Journal

Prof. Campbell holds a position as the co-Editor in Chief. He occasionally reimburses small expenses related to Journal’s work, including travel and consumables.

### Unpaid positions

Member, MRC Africa Research Excellence Fund (AREF), College of Experts, since 2016;
Member, WHO PIP burden of disease advisory group, since 2015;
Member of international Scientific Advisory Group, INDEPTH network, since 2014;
Member of Expert Advisory Group for “The Making of Genomic Medicine Wellcome Trust Programme; University of Edinburgh), since 2013;
Member, Public Health, Health Services Research and Primary Care (A; subpanel 2) Research Excellence Framework (REF), 2018-2021;
Numerous technical advisor appointments to WHO in the past 5 years.
